# Who’s Going to Manage the Thyroid Cancer?

**DOI:** 10.1210/clinem/dgaa514

**Published:** 2020-08-10

**Authors:** Diana J Chang, Angela M Leung

**Affiliations:** 1 Division of Endocrinology, Diabetes, and Metabolism; Department of Medicine; UCLA David Geffen School of Medicine, Los Angeles, California; 2 Division of Endocrinology, Diabetes, and Metabolism; Department of Medicine; VA Greater Los Angeles Healthcare System, Los Angeles, California

**Keywords:** thyroid cancer, primary care physicians, endocrinologists, health services

The number of thyroid cancer survivors has increased substantially in the past few decades, a trend that has been due in part to the increasing incidence of thyroid cancer, a high 5-year relative survival rate of individuals with differentiated thyroid cancer, and the relatively young age of many patients at diagnosis (1, 2). The need for long-term monitoring and surveillance for recurrent thyroid cancer can be a challenge if performed by endocrinologists alone. Typical follow-up care for patients with differentiated thyroid cancer consists of obtaining periodic serum thyroglobulin levels, neck ultrasounds, and levothyroxine adjustment for suppression therapy as needed (3). Thus far, there is limited literature on the role of primary care physicians in the follow-up care of thyroid cancer patients. The recent study by Radhakrishnan et al is the first to address this issue by understanding the primary care physician’s extent of involvement and confidence in handling survivorship care of patients with differentiated thyroid cancer (4), a condition with a usually good prognosis and relatively low risk of recurrence.

Interestingly, the study’s findings showed that 76% of primary care physicians reported being involved in their patients’ long-term thyroid cancer surveillance, with only 5% reporting that they are never involved. However, despite the vast majority of primary care providers reporting the management of patients with thyroid cancer in their practice, less than 50% were highly confident in discussing many aspects of thyroid cancer follow-up care. In addition, 54% of primary care physicians noted that clinical guidelines are their most influential knowledge source in treating thyroid cancer, with only 22.5% citing their training in medical school and residency (4). As such, internal medicine curricula may not be adequately preparing primary care physicians in this field. Less than 2% of questions on the current certifying examination of the American Board of Internal Medicine are devoted to thyroid disorders, including thyroid cancer (5). Therefore, improved training and educational efforts are necessary in this area before considering the transition of long-term thyroid cancer care to the primary care setting.

The authors surveyed a relatively diverse sample of primary care physicians; one-third were nonwhite and 74% were from private practice settings. However, the results of this study may not be generalizable to all primary care physicians because the survey was conducted of US providers in the state of Georgia and the metropolitan area of Los Angeles. Both are large, urban cities with dense populations that likely have a greater number of and ease of referral to endocrinologists compared to their rural counterparts. Primary care physicians in less populated areas may be asked to monitor their thyroid cancer patients given more limited access to subspecialists. McDow and colleagues showed that the 5-year and 10-year survival rates of thyroid cancer are significantly lower in rural areas, compared to those in urban counties, although it is unclear whether these differences are due to health care disparities, differences in therapy, or other reasons (6). Primary care physicians in more rural areas likely play a central role in thyroid cancer survivorship, and it would be of interest to also survey physician perspectives and experiences in these groups.

Since the US Institute of Medicine released its 2006 landmark report on transitioning survivorship care of all cancer types to primary care providers (6), there has been growing research focused on understanding how primary care clinicians perceive their role in delivering care to patients with a history of cancer (7). There continue to be divergent views, mostly in regards to breast, prostate, and colon cancers, with some clinicians inclined to have these patients be managed by subspecialists, whereas others support transitioning the majority of long-term cancer care to the primary care setting. The study by Radhakrishnan and colleagues also reflected these opposing opinions—53% of primary care physicians believe they should be responsible for continuing thyroid cancer care after the patient’s initial treatment (4).

As US health care costs continue to rise in the setting of limited resources, future research should focus on establishing strategies for developing high-quality, team-based care for the increasing number of thyroid cancer patients requiring long-term surveillance. A shared decision model between primary care physicians, endocrinologists, and other important members of the care team seems critical at the outset of care, one that defines specific roles for each member and allows management to transition back and forth between the specialist and primary care doctor as needed.

## Data Availability

Data sharing is not applicable to this article because no data sets were generated or analyzed during the present study.

## Additional Information

***Disclosure Summary:*** None declared.
